# A New Model for Predicting Nonsentinel Lymph Node Metastasis in Early-Stage Breast Cancer Using MMP15

**DOI:** 10.1155/2022/8675705

**Published:** 2022-08-18

**Authors:** Xue Zeng, Yubing Li, Chaonan Sun, Zhuang Liu, Jiaming Zhao, Xinchi Ma, Yanyu Zhang, Na Zhang

**Affiliations:** Department of Radiation Oncology, Cancer Hospital of China Medical University, Liaoning Cancer Hospital & Institute, Cancer Hospital of Dalian University of Technology, Shenyang 110042, China

## Abstract

**Background:**

In early-stage breast cancer (BC) patients, 40–70% of lymph node metastases are limited to the sentinel lymph nodes (SLNs). Patients at low risk for nonsentinel lymph node (NSLN) metastasis should be exempt from axillary lymph node dissection (ALND) or regional lymph node radiotherapy (RNI).

**Methods:**

The present study included 237 female early-stage BC patients with positive SLNs who received ALND. Based on the clinicopathological factors of the 158 patients in the training cohort, multivariate analysis was used to determine the independent risk factors for NSLN metastasis, which were used to establish the NSLN metastasis prediction model. The calibration and discrimination of this model were tested with the training and validation cohorts and compared to the Memorial Sloan Kettering Cancer Center (MSKCC) model.

**Results:**

Tumor size, neural invasion, lymphovascular invasion, expression of matrix metalloproteinase 15 (MMP15) in the cytoplasm, and the number of positive SLNs were statistically significant by multivariate analysis (*P* < 0.05), which were used to establish the new model. The MSKCC model was verified by the training cohort, and the area under the receiver-operating characteristic (ROC) curve was 0.733 (95% CI: 0.650–0.816), which was less than that of the new model (0.824; 95% CI: 0.760–0.889). The area under the ROC curve in the validation cohort for the new model was 0.773 (95% CI: 0.669–0.877), and the calibration performed well. The false-negative rates were 3.2%, 6.5%, and 14.5% for the predicted probability cut-offs of 50%, 60%, and 70%, respectively.

**Conclusions:**

The new model included five variables: tumor size, neural invasion, lymphovascular invasion, cytoplasmic MMP15 expression, and the number of positive SLNs. The model with a cut-off of 60% could accurately identify low-risk patients with NSLN metastasis.

## 1. Introduction

Sentinel lymph node biopsies (SLNB) can accurately judge the status of axillary lymph nodes in patients with early-stage breast cancer (BC), and patients with negative sentinel lymph nodes (SLNs) can be exempt from axillary lymph node dissection (ALND) and other local treatments [1–3]. When the SLNs are positive, the situation is complicated; the choice of SLNB only, supplementary ALND, or regional lymph node radiotherapy (RNI) is controversial. Studies have shown that there is no significant difference in disease-free survival (DFS) and total survival (OS) between SLN micrometastases or SLN1-2 macrometastases with SLNB only or RNI compared to supplementary ALND [4–10]. Therefore, patients with limited axillary lymph node metastasis, who meet the above criteria, can be exempt from ALND.

Studies have shown that 40–70% of lymph node metastases in patients with early-stage BC are limited to the sentinel lymph nodes [11]. Thus, subsequent local treatment may lead to overtreatment. The nonsentinel lymph node (NSLN) metastasis prediction model established by Memorial Sloan Kettering Cancer Center (MSKCC) can help to determine whether patients exempt from ALND require RNI at a later date [12]. When the model determines that NSLN metastasis is a low risk, patients do not need RNI. Other cancer centers found the area under the receiver-operating characteristic (ROC) curve for the MSKCC model to be from 0.54 to 0.86 [12, 13] (i.e., the range varied greatly), and the diagnostic value was lower when the area under the model ROC curve was less than 0.7. Thus, the MSKCC model is not generally applicable. The detection of molecular markers related to the treatment and prognosis of BC is the cornerstone for accurate treatment. A combination of gene expression and clinicopathological factors could identify more low-risk patients and improve the sensitivity and specificity of the model. However, previous studies have not demonstrated the role of molecular markers in predicting NSLN metastasis in detail. Therefore, we included molecular indicators that affect BC biology in the present study combined with the molecular level to explain the relationship between these variables and NSLN status.

The 28-gene detection model is based on Asian genes and can simultaneously evaluate the risk of local recurrence and distant metastasis in BC. BC patients with axillary lymph node N1-2 can be divided into low-risk and high-risk groups. Patients with a low risk of local recurrence can consider waiving RNI. The 28-gene detection model includes 18 core genes and ten auxiliary genes, of which the invasion gene MMP15 is considered a core gene [14]. Data on the role of MMP15 in cancer deserve special attention. MMP15 can affect the tumor microenvironment by degrading the extracellular matrix and affecting signal transduction by interacting with growth factors. MMP15 plays a role in invasion, angiogenesis, and the formation of premetastatic tumor niches [15, 16]. Changes in MMP15 expression levels have been described in various cancers, including BC [17–22]. MMP15 promotes the invasion of transformed cells due to the degradation of the extracellular matrix. In addition, MMP15 can disrupt cell-cell contacts and activate cell motility and migration.

At present, there is no study on the expression of MMP15 in metastatic lymph nodes but increased MMP15 expression was strongly associated with lymph node involvement [23]. Combined with the biological characteristics of MMP15, we inferred that high MMP15 expression would be positively correlated with lymph node metastasis in BC. As the first lymph nodes to receive lymph from tumors, SLNs are maximally exposed to tumor-derived bioactive molecules. And the biological expression of SLNs is the most direct factor to judge the metastasis of NSLN [24–26]. Therefore, the expression of MMP15 in SLNs could be used as a risk factor for predicting NSLN metastasis. By combining MMP15 expression with molecular typing [24, 27, 28], inflammatory indicators [29], and other factors related to NSLN metastasis, we established a new prediction model with higher accuracy that is more suitable for the Chinese population.

## 2. Methods

### 2.1. Patients

A total of 237 female early-stage BC patients in Liaoning Cancer Hospital were enrolled in this study from June 2015 to December 2019 based on the following inclusion criteria: clinical stage T1 to 2N0M0; ≥3 SLNs were detected; SLN metastases were confirmed by pathology; ALND was performed; postoperative pathology was confirmed as invasive BC; complete case information was available. The exclusion criteria included adjuvant therapy before surgery, bilateral or metastatic BC, or previous history of other cancers. MMP15 expression was determined by immunohistochemistry. Based on the order in which they underwent surgery, the patients were divided into training and validation cohorts, with 158 patients assigned to the training cohort and 79 patients to the validation cohort. The research scheme was approved by the Ethics Committee of Liaoning Cancer Hospital.

### 2.2. Sentinel Lymph Node Biopsy (SLNB)

The *Guidelines and Norms for Diagnosis and Treatment of Breast Cancer of the China Anti-Cancer Association (2019)* recommend the combined use of blue dye and radionuclide tracer to increase the success rate of SLNB and reduce the false-negative rate [30]. Due to issues with the accessibility to the radionuclide tracer, we used methylene blue as the tracer. Although the single staining method with blue dye was considered a feasible and acceptable accuracy tracer [31], our study still required patients to have at least three SLNs since the significant correlation between the number of detected SLNs and the false-negative rate of SLNB has been confirmed by some studies [32, 33]. With the increase in the number of detected SLNs, the false-negative rate of SLNB decreases significantly, and the detection of ≥3 SLNs is considered a standard operation in the clinic. Methylene blue (2 mL) was injected into the areola or around the tumor for 10 to 15 min. The fat and connective tissue were cut along the lateral edge of the pectoralis major muscle and separated layer by layer. The first blue-stained lymph node on the lymphatic vessel was the SLN. The enlarged hard axillary lymph nodes found by the surgeons were also regarded as SLNs and sent for a rapid intraoperative frozen histopathological examination. If an SLN was positive, ALND was performed at the same time. If the SLN was negative, a routine pathological examination of the section was performed after the operation before determining whether further treatment was necessary. No ALND or other follow-up treatment was performed unless the routine pathology showed a positive SLN.

### 2.3. Pathological Diagnosis of Postoperative Lymph Nodes

The SLNs were divided into at least two tissue blocks along the long axis. One block was cut into one section for rapid intraoperative frozen histopathological examination. For routine pathological examination, the remaining tissue was fixed with 10% formaldehyde and embedded in paraffin. Each piece was cut into a 5-*μ*m-thick section and stained with hematoxylin and eosin. All NSLNs were processed in the same manner. The diagnosis was made by two pathologists. Positive lymph nodes included micrometastases and macrometastases. A micrometastasis was defined as a tumor in the lymph node with a maximum diameter from >0.2 mm to ≤2.0 mm or more than 200 tumor cells on the section of a lymph node. A macrometastasis was defined as a tumor with a maximum diameter > 2.0 mm [34].

### 2.4. MMP15 Expression in Positive SLNs

MMP15 expression was evaluated in paraffin-embedded SLNs using immunohistochemistry. Sections (3 *μ*m) were deparaffinized and hydrated, and then, antigen retrieval was carried out at high temperature and pressure. The sections were immersed in 3% hydrogen peroxide for 15 min to block the endogenous peroxidase activity. All sections were incubated with one or two drops of primary MMP15 antibody (rabbit polyclonal antibody, Abcam, ab15475, 1/100) for 60 min at room temperature. Subsequently, the sections were incubated with one or two drops of horseradish peroxidase-labeled secondary antibody (ready-to-use second-generation Elivision Plus broad-spectrum immunohistochemical kit) for 30 min at room temperature. The sections were treated with DAB chromogenic solution for 1 to 4 min, and then, the samples were rinsed with tap water. Finally, the sections were counterstained with hematoxylin solution for 1 to 5 min.

MMP15 expression was scored based on the staining intensity and the percentage of positive cells. The staining intensity was scored as follows: 0, colorless; 1, light yellow; 2, dark yellow; and 3, brown. Then scoring for the percentage of positive cells was as follows: 0, negative; 1, ≤10%; 2, 11–50%; 3, 51–75%; and 4, >75%. The MMP15 expression score was the product of the staining intensity and the percentage of positive cells. A score > 3 indicated the sample was MMP15 positive [35].

### 2.5. Statistical Analysis

Statistical analysis was performed using IBM SPSS Statistics version 24. To compare the clinical and pathological characteristics of the patients in the training and validation cohorts, the chi-square test or Fisher's exact test was used for categorical variables and the Mann–Whitney *U* test was used for continuous variables. The clinical and pathological data related to NSLN metastasis in the training cohort were analyzed by univariate and multivariate logistic regression. The independent risk factors obtained from the multivariate analysis were used to construct the NSLN metastasis risk prediction model. A *P* value < 0.05 indicated statistical significance.

R language (version 3.6.1, Copyright (C) 2019, The *R* Foundation for Statistical Computing) was used to draw the nomogram and calibration plot. The calibration and discrimination of the model were tested by drawing the calibration plot and calculating the area under the ROC curve to complete the internal verification of the training cohort. The validation cohort was used to test the new model as an external verification. In addition, the ability of the MSKCC model to predict NSLN metastasis risk was evaluated in the training cohort using the MSKCC online prediction tool (http://nomograms.mskcc.org/breast/BreastAdditionalNonSLNMetastasesPage.aspx). After inputting the corresponding clinicopathological indicators, the probability of NSLN metastasis was obtained.

## 3. Results

### 3.1. Demographics and Clinicopathological Characteristics

**Figure 1:**
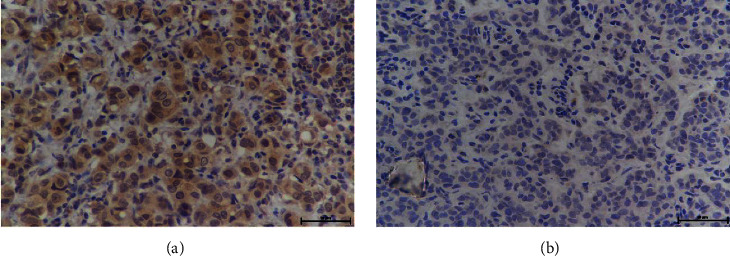
The expression of matrix metalloproteinase 15 (MMP15) in the cytoplasm of positive sentinel lymph nodes (SLNs); the cells were magnified 400 times. (a) The expression of MMP15 was positive. (b) The expression of MMP15 was negative.

BC patients in Liaoning Cancer Hospital were screened using the hospital information system from June 2015 to December 2019. A total of 237 patients met the inclusion criteria, with 47 undergoing breast-conserving treatment and 190 undergoing mastectomy. The patients were divided chronologically into training (158) and validation (79) cohorts. A total of 1,118 SLNs were detected in the 237 patients (average 5, median 4, range 3–12). There were 17 patients with micrometastases (7.2%) and 220 patients with macrometastases (92.8%). The NSLN metastasis rate was 38.0% (90/237). MMP15 expression in the positive SLNs is presented in Figures 1(a) and 1(b). There were differences in tumor size and histological grade between the training and validation cohorts (*P* < 0.05). The other characteristics were comparable between the two cohorts (Table 1).

### 3.2. Univariate and Multivariate Analysis Related to NSLN Metastasis

The training cohort was stratified based on the presence or absence of NSLN metastasis. The NSLN metastasis rate was 39.2% (62/158). Factors included in the analysis were patient age, tumor size, tumor type and histological grade, size of the SLN metastasis, tumor quadrant, the number of positive SLNs, the number of negative SLNs, the percentage of positive SLNs, MMP15 expression in the cytoplasm, MMP15 expression in the nucleus, molecular subtype, Ki-67 status, lymphovascular invasion, neural invasion, multifocality, and inflammation indicators (e.g., the ratio of the absolute number of platelets to the absolute number of lymphocytes (PLR); the ratio of the absolute number of neutrophils to the absolute number of lymphocytes (NLR); the ratio of the absolute number of neutrophils to the absolute number of monocytes (NMR]); and blood cell counts from retrospective data collected within one week before surgery). The results of univariate and multivariate logistic regression analysis are shown in Table 2 and 3. Tumor size, lymphovascular invasion, neural invasion, MMP15 expression in the cytoplasm, the number of positive SLNs, the number of negative SLNs, the percentage of positive SLNs, and NLR were statistically significant in univariate regression analysis (*P* < 0.05). Tumor size, neural invasion, lymphovascular invasion, MMP15 expression in the cytoplasm, and the number of positive SLNs were statistically significant in multivariate regression analysis (*P* < 0.05) and independent risk factors for NSLN metastasis.

### 3.3. Comparison of the MSKCC Model and New Model and Validation of the New Model

**Figure 2:**
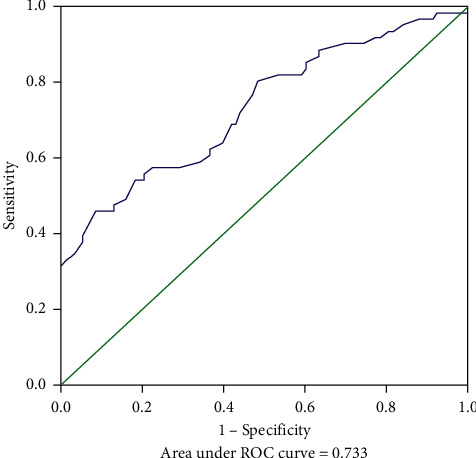
Receiver operating characteristic (ROC) curve calculation for the MSKCC model applied to the training cohort (*n* = 154).

**Figure 3:**
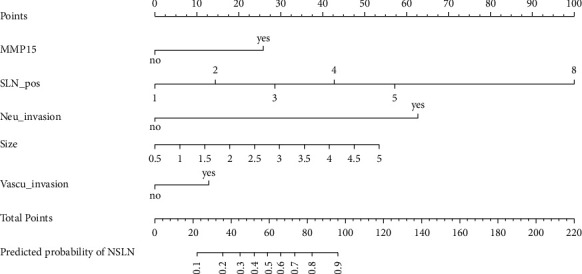
Nomogram to predict the probability of nonsentinel lymph node (NSLN) metastasis in sentinel lymph nodes (SLNs)-positive breast cancer patients. The new model included five variables: matrix metalloproteinase 15 (MMP15) expression in the cytoplasm, number of positive SLNs, neural invasion, tumor size, and lymphovascular invasion. After each variable was assigned, it corresponded to the score of the first row in the nomogram and then the five scores were summed to get the total score. From this score, the NSLN metastasis probability could be obtained.

**Figure 4:**
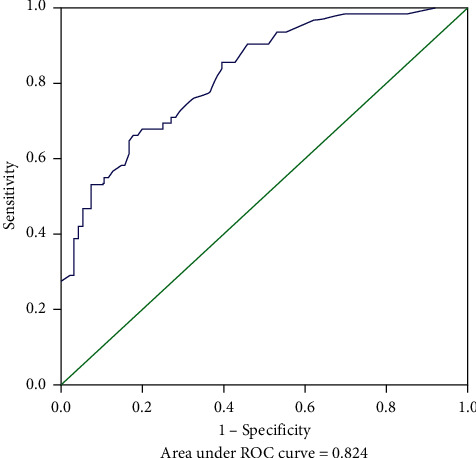
Receiver operating characteristic (ROC) curve calculation for the new model applied to the training cohort (*n* = 158).

**Figure 5:**
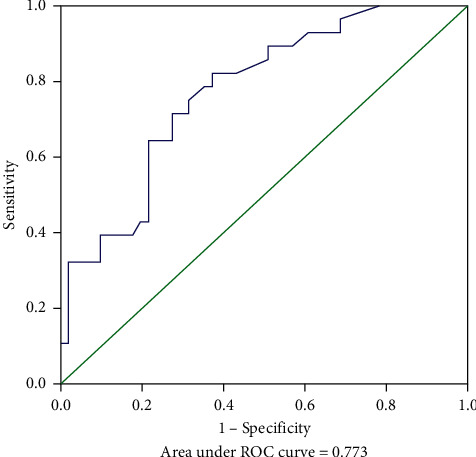
Receiver operating characteristic (ROC) curve calculation for the new model applied to the validation cohort (*n* = 79).

**Figure 6:**
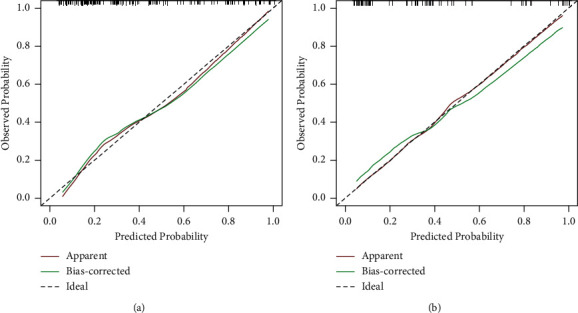
Calibration plot for the new model—the actual versus the predicted probabilities of nonsentinel lymph node (NSLN) metastasis. The trend lines of the (a) training and (b) validation cohorts differed only slightly, indicating that there was a good match between the observation and prediction results.

The MSKCC model was verified using the training cohort (154 patients were applicable to the MSKCC model), with an area under the ROC curve of 0.733 (95% CI: 0.650–0.816) (Figure 2), which was smaller than the area under the ROC curve for the original MSKCC model (0.760). We used the independent risk factors for NSLN metastasis from the multivariate logistic regression analysis to establish a new NSLN metastasis prediction model. The nomogram for this model is presented in Figure 3. The new model included five variables (tumor size, number of positive SLNs, lymphovascular invasion, neural invasion, and MMP15 expression in the cytoplasm). After each variable was assigned, it corresponded to the score of the first row in the nomogram and then the five scores were summed to get the total score. From this score, the NSLN metastasis probability could be obtained. The areas under the ROC curve of the new model were 0.824 (95% CI: 0.760–0.889; Figure 4) and 0.773 (95% CI: 0.669–0.877; Figure 5) in the training and validation cohorts, respectively. The calibration plot showed a good match between the observation and prediction results of the training and validation cohorts (Figures 6(a) and 6(b)). The logistic regression model for predicting NSLN metastasis is as follows: logit (p) = −2.853 + 1.592*∗* positive expression of MMP15 in cytoplasm + 3.712*∗* neural invasion + 0.898*∗* lymphovascular invasion + 0.641*∗* tumor size + 0.904*∗* the number of positive SLNs.

### 3.4. Comparison of Different Cut-Off Prediction Capabilities

To evaluate the ability of the model to screen low-risk patients, cut-offs of 50%, 60%, and 70% were used, and the false-negative rates were 3.2%, 6.5%, and 14.5%, respectively. A cut-off of 60% was most appropriate. The new model covered 49 patients (31.0%), including four patients with NSLN metastases. The specificity was 46.9%, the negative predictive value was 91.8%, and the total coincidence rate was 65.2% (Table 4).

## 4. Discussion

When SLNs are positive, only half of the patients with NSLN metastases can benefit from ALND or RNI. The NSLN metastasis risk prediction model presented in the current study could help determine whether NSLN metastases are present in early-stage BC patients. The NSLN metastasis probability is calculated by substituting a patient's clinicopathological factors into the corresponding variables of the model. If the probability is less than the preset cut-off, then no NSLN metastases are present. This information can help patients avoid local overtreatment and reduce the incidence of complications caused by various treatments. The NSLN metastasis risk prediction model could improve the quality of life of patients and fully reflect the concept of individualized treatment.

Two studies (IBCSG23-01 and AATRM) found the NSLN metastasis rate in early-stage BC with SLN micrometastases to be 13%. There were no significant differences in the DFS and OS between the SLNB and ALND groups, and the incidences of upper limb sensory abnormalities, motor neuropathy, and lymphedema in the ALND group were high [4–6]. The ACOSOG Z0011 study showed that patients with SLN1–2 macrometastases and clinical stage of T1–2N0M0 received whole-breast radiotherapy and systemic therapy after breast-conserving surgery and could avoid ALND. In the present study, the NSLN metastasis rate was 27.3%. The 10-year OS and DFS of the SLNB group were not inferior to those of the ALND group [7, 8]. As a prospective, randomized, multicenter trial, ACOSOG Z0011 has been accepted by more and more scholars for its significance in changing clinical practice despite its shortcomings. Breast-conserving surgery became popular in the mid-1990s, and it is used for about 20% of early-stage BC cases in China from 1999 to 2013 [36]. Patients who have undergone mastectomy are more likely to choose ALND, so the NSLN metastasis risk prediction model is of more reference significance for patients undergoing mastectomy. In the ACOSOG Z0011 study, 52.6% and 50% of the SLNB and ALND groups received high tangent field radiotherapy, respectively; 16.9% and 21.2% received RNI, respectively. The analysis showed that radiotherapy played an important role in reducing the recurrence rate in the axillary lymph nodes [37, 38]. The 5-yearfollow-up results for the AMAROS and OTOASOR studies showed that the DFS and OS of RNI in early-stage BC patients with SLN1-2 positive were not inferior to those of ALND. The NSLN metastasis rates of the two studies were 33% and 38.5%, respectively [9, 10]. These data indicate that patients with SLN micrometastases (>2 mm) or SLN1-2 macrometastases (>2 mm) can be exempt from ALND. Moreover, these data combined with NSLN metastasis risk prediction model can better guide clinical practice. Namely, patients at low risk for NSLN metastasis can be exempt from ALND and other local treatments, whereas high-risk patients can be considered for radiotherapy instead of ALND.

In previous studies, the variables contained in the NSLN metastasis risk prediction model were traditional clinicopathological factors, and the model discrimination was not high. In contrast, our study combined these factors with molecular indicators affecting BC biology to develop a new model with higher accuracy. Our study observed MMP15 expression in positive SLNs of BC patients and found a significant correlation between high MMP15 expression in the cytoplasm and NSLN metastasis. The positive MMP15 expression in the cytoplasm was an independent predictor of NSLN metastasis. This study confirmed the significance of MMP15 in the growth, invasion, and metastasis of BC both from a clinical and biological standpoint.

The probability of NSLN metastasis calculated by the model itself could not determine a treatment method. Thus, the best cut-off between high and low risk must be established to ensure the efficiency of the model. If the model is designed to screen low-risk patients, the optimal cut-off can be determined by indicators, such as a negative predictive value, false-negative rate, and specificity, so that the model can cover more patients. A false-negative rate of 10% or less is widely accepted [25, 39]. If the model is used to screen high-risk patients, attention should be paid to the positive predictive value, false-positive rate, and sensitivity.

Compared to the MSKCC model, the new model contained fewer variables and was more convenient and efficient in its clinical application. In the same population, the area under the ROC curve of the new model was 0.824, which was higher than that of the MSKCC model (0.733). It also had a good calibration. Although the new model performed well in the validation cohort, its population came from our hospital and had limited extension. Therefore, the new model needs further verification using datasets from other centers before formally applying it in the clinic.

## Figures and Tables

**Table 1:** Comparison of clinicopathological characteristics of the training cohort and validation cohort.

Variables	Total	Training *n* (%)	Validation *n* (%)	*P* value
No. of cases	237	158	79	

Age (years)				0.782^*∗*^
Median	49	49	49	
≤50	129	85 (53.8)	44 (55.7)	
>50	108	73 (46.2)	35 (44.3)	

Lymphovascular invasion				0.234^*∗*^
No	181	117 (74.1)	64 (81.0)	
Yes	56	41 (25.9)	15 (19.0)	

Neural invasion				1.000^*∗*^
No	223	149 (94.3)	74 (93.7)	
Yes	14	9 (5.7)	5 (6.3)	

Expression of MMP15 in cytoplasm				0.637^*∗*^
Negative	145	95 (60.1)	50 (63.3)	
Positive	92	63 (39.9)	29 (36.7)	

Expression of MMP15 in nucleus				0.386^*∗*^
Negative	210	142 (89.9)	68 (86.1)	
Positive	27	16 (10.1)	11 (13.9)	

Size of the SLN metastasis				0.722^*∗*^
Micrometastasis	17	12 (7.6)	5 (6.3)	
Macrometastasis	220	146 (92.4)	74 (93.7)	

NSLN metastasis				0.570^*∗*^
No	147	96 (60.8)	51 (64.6)	
Yes	90	62 (39.2)	28 (35.4)	

Multifocality				1.000^*∗*^
No	225	150 (94.9)	75 (94.9)	
Yes	12	8 (5.1)	4 (5.1)	

Tumor type				0.722^*∗*^
Invasive ductal carcinoma	220	146 (92.4)	74 (93.7)	
Others^†^	17	12 (7.6)	5 (6.3)	

Tumor size				0.001^*∗*^
≤2 cm	169	102 (64.6)	67 (84.8)	
>2 cm	68	56 (35.4)	12 (15.2)	

Tumor quadrant				0.269^*∗*^
Outer upper	122	86 (54.4)	36 (45.6)	
Others	113	72 (45.6)	41 (51.9)	

Molecular subtype				0.495^*∗*^
Luminal A	47	34 (21.5)	13 (16.5)	
Luminal B	156	105 (66.5)	51 (64.6)	
HER2 overexpression	10	7 (4.4)	3 (3.8)	
Triple negative	14	7 (4.4)	7 (8.9)	

Ki-67 status				0.118^*∗*^
≤0.2	116	83 (52.5)	33 (41.8)	
>0.2	121	75 (47.5)	46 (58.2)	

Histological grade				0.002^*∗*^
I–II	184	116 (73.4)	68 (86.1)	
III	32	29 (18.4)	3 (3.8)	

Number of positive SLNs				0.741^*∗*^
1	96	61 (38.6)	35 (44.3)	
2	84	56 (35.4)	28 (35.4)	
3	37	27 (17.1)	10 (12.7)	
4	10	6 (3.8)	4 (5.1)	
≥5	10	8 (5.1)	2 (2.5)	

Number of negative SLNs				0.236^*∗*^
0	17	10 (6.3)	7 (8.9)	
1	45	36 (22.8)	9 (11.4)	
2	62	36 (22.8)	26 (32.9)	
3	45	31 (19.6)	14 (17.7)	
4	32	20 (12.7)	12 (15.2)	
≥5	36	25 (15.8)	11 (13.9)	

Percentage of positive SLNs				0.354^*∗∗*^
Mean ± standard deviation	45% ± 25%	46% ± 25%	43% ± 25%	

PLR				0.361^*∗∗*^
Mean ± standard deviation	140.44 ± 46.84	137.66 ± 43.79	145.98 ± 52.20	

NLR				0.794^*∗∗*^
Mean ± standard deviation	2.09 ± 0.91	2.08 ± 0.84	2.11 ± 1.05	

NMR				0.769^*∗∗*^
Mean ± standard deviation	12.47 ± 3.89	12.46 ± 3.78	12.50 ± 4.10	

^*∗*^ *P* value by the chi-square test or Fisher's exact test; ^*∗∗*^*P* value by the Mann-–Whitney *U* test; ^†^Invasive lobular carcinoma, mixed carcinoma (invasive ductal carcinoma and invasive lobular carcinoma), tubular carcinoma, mucinous adenocarcinoma, papillary carcinoma, and sweaty adenoid carcinoma; SLNs, sentinel lymph nodes; NSLN, nonsentinel lymph node; MMP15, matrix metalloproteinase 15; PLR, the ratio of the absolute number of platelets to the absolute number of lymphocytes; NLR, the ratio of the absolute number of neutrophils to the absolute number of lymphocytes; NMR, the absolute number of neutrophils to the absolute number of monocytes.

**Table 2:** Univariate logistic regression analysis of factors related to NSLN metastases.

Variables	Univariate analysis		95% CI	
158	*P* value	HR	Lower	Upper
Age (years)				
>50 versus ≤50	0.833	0.933	0.492	1.772

Tumor type				
Others^†^ versus invasive ductal carcinoma	0.664	0.759	0.218	2.635
Tumor size (cm)	0.005	1.784	1.19	2.675

Histological grade				
III versus I–II	0.552	0.773	0.33	1.809

Lymphovascular invasion				
Yes versus no	0.011	2.556	1.236	5.286

Neural invasion				
Yes versus no	0.014	14.074	1.714	115.567

Size of the SLN metastasis				
Macrometastasis versus micrometastasis	0.115	3.488	0.738	16.494

Expression of MMP15 in cytoplasm				
Positive versus negative	0.006	2.503	1.294	4.842

Expression of MMP15 in nucleus				
Positive versus negative	0.356	1.63	0.578	4.596
Number of positive SLNs	≤0.001	2.153	1.5	3.09
Number of negative SLNs	0.002	0.718	0.58	0.888

Molecular subtype				
Luminal B versus luminal A	0.248	3.714	0.401	34.443
HER2 overexpression versus luminal A	0.207	4	0.465	34.432
Triple negative versus luminal A	0.116	8	0.598	106.936
Ki-67 status				
>0.2 versus ≤0.2	0.137	1.629	0.856	3.099

Tumor quadrant				
Outer upper versus others	0.288	0.704	0.369	1.344

Multifocality				
Yes versus no	0.526	1.586	0.382	6.591
Percentage of positive SLNs	≤0.001	20.008	4.736	84.523
PLR	0.407	0.997	0.989	1.004
NLR	0.033	0.619	0.398	0.963
NMR	0.057	0.917	0.838	1.003

^†^Invasive lobular carcinoma, mixed carcinoma (invasive ductal carcinoma and invasive lobular carcinoma), tubular carcinoma, mucinous adenocarcinoma, papillary carcinoma, and sweaty adenoid carcinoma; SLNs, sentinel lymph nodes; MMP15, matrix metalloproteinase 15; PLR, the ratio of the absolute number of platelets to the absolute number of lymphocytes; NLR, the ratio of the absolute number of neutrophils to the absolute number of lymphocytes; NMR, the absolute number of neutrophils to the absolute number of monocytes.

**Table 3:** Multivariate logistic regression analysis of factors related to NSLN metastases.

Variables	*P* value	OR	95% CI	
			Lower	Upper
Tumor size (cm)	0.011	1.899	1.157	3.117

Lymphovascular invasion				
Yes versus no	0.048	2.455	1.008	5.981

Neural invasion				
Yes versus no	0.003	40.935	3.665	457.198

Expression of MMP15 in cytoplasm				
Positive versus negative	≤0.001	4.912	2.083	11.585
Number of positive SLNs	0.038	2.47	1.054	5.789
Number of negative SLNs	0.483	0.825	0.481	1.413
Percentage of positive SLNs	0.714	0.361	0.002	84.504
NLR	0.124	0.64	0.362	1.131

SLNs, sentinel lymph nodes; MMP15, matrix metalloproteinase 15; NLR, the ratio of the absolute number of neutrophils to the absolute number of lymphocytes.

**Table 4:** The ability of the new model to screen low-risk NSLN metastases with a cut-off of 50%, 60%, or 70%.

Cut-off point	Covered patients	Number of patients with NSLN metastasis	Specificity (%)	False-negative rate (%)	Negative predictive value (%)	Total coincidence rate (%)
≤50%	37	2	36.5	3.2	94.6	60.1
≤60%	49	4	46.9	6.5	91.8	65.2
≤70%	66	9	59.4	14.5	86.4	69.6

NSLN, nonsentinel lymph node.

## Data Availability

All data generated or analyzed during this study are included in this article.
